# Efficacy and safety of metformin in the treatment of gestational diabetes: A protocol for systematic review and meta-analysis

**DOI:** 10.1097/MD.0000000000023954

**Published:** 2021-01-08

**Authors:** Weirong Mao, Shengzhi Zhang, Lanying Wang, Shaohua Shen

**Affiliations:** Yuyao People's Hospital, Yuyao, Zhejiang province, China.

**Keywords:** gestational diabetes, metformin, protocol, randomized controlled trial, systematic review

## Abstract

**Background:**

The incidence of gestational diabetes is increasing, which not only cause adverse pregnancy outcomes, but also increases the risk of diabetes for pregnant women and their children. Insulin is the gold standard for the treatment of gestational diabetes, but there are some disadvantages, such as poor patient compliance. Metformin has been used in the treatment of gestational diabetes, but the evaluation of its efficacy and safety is lack of reliable evidence-based medicine evidence. The purpose of this study was to systematically investigate the efficacy and safety of metformin in the treatment of diabetic gestational diabetes.

**Methods:**

Computer searches China National Knowledge Infrastructure, Wanfang, Vipu Information Chinese Journal Service Platform and China Biomedical Database, PubMed, Embase, Web of Science, the Cochrane Library from the establishment of the database to November 2020, randomized controlled clinical trials of metformin in the treatment of gestational diabetes mellitus were conducted in English and Chinese. Two researchers independently carried out data extraction and literature quality evaluation on the quality of the included study, and the included literature was analyzed by Meta using RevMan5.3 software.

**Results:**

In this study, the efficacy and safety of metformin in the treatment of diabetic gestational diabetes were investigated by evaluating the outcome indicators of pregnant women and newborn babies respectively.

**Conclusion:**

This study will provide reliable evidence for the clinical application of metformin in the treatment of diabetic gestational diabetes.

**Ethics and dissemination:**

The private information from individuals will not be published. This systematic review also will not involve endangering participant rights. Ethical approval is not required. The results may be published in a peer-reviewed journal or disseminated in relevant conferences.

**OSF Registration number:**

DOI 10.17605/ OSF.IO / 7RB95

## 1 Introduction

Gestational Diabetes Mellitus (GDM) refers to diabetes or impaired glucose tolerance found for the first time during pregnancy.^[1]^ With the improvement of people's living standards, the incidence of obesity and overweight is increasing,^[2]^ and the incidence of GCM in all regions has an upward trend. According to statistics, the global average incidence of GDM is 7.0%,^[3]^ 9.3% to 18.9%^[4]^ in China, and 24.3% in Riyadh, the capital of Saudi Arabia.^[5]^ GDM is seriously harmful to people's health, It not only causes adverse pregnancy outcomes such as macrosomia, preeclampsia, dystocia and neonatal hyperbilirubinemia during pregnancy, but also increases the risk of metabolic diseases such as type 2 diabetes mellitus in mothers and their children after pregnancy, resulting in a huge long-term economic burden on the state, society and families.^[7]^ Patients with GDM have a higher risk of metabolic dysfunction in the future and after 15 to 25 years, the risk is as high as 50% to 70%.^[8]^

At present, the main therapeutic drugs for GCM are insulin and oral hypoglycemic drugs, and insulin is the first choice for the treatment of GDM,^[9]^ due to the way of administration and inconvenient storage, oral hypoglycemic drugs are more acceptable to patients. Metformin, as a first-line oral hypoglycemic drug for the treatment of type 2 diabetes, has the advantages of convenient oral administration, simple storage and low price. However, metformin can pass through the fetal barrier, so whether the treatment of GDM causes adverse outcomes has not been clarified, and its safety is still controversial.^[10]^ At present, a number of randomized controlled trials have confirmed that metformin has the advantages of low cost and ease of use in the treatment of GDM, and can reduce weight gain during pregnancy, but does not increase the adverse outcome compared with insulin.^[11–13]^Due to the differences in research protocols and curative effect among clinical trials, the differences in research results have influenced the promotion of this therapy to some extent. Therefore, this study plans to systematically evaluate the efficacy and safety of metformin in the treatment of gestational diabetes, in order to provide an evidence-based basis for the treatment of GDM.

## 2 Methods

### 2.1 Protocol register

This protocol of systematic review and meta-analysis has been drafted under the guidance of the preferred reporting items for systematic reviews and meta-analyses protocols (PRISMA-P). Moreover, it has been registered on open science framework on November 27, 2020. (Registration number: DOI 10.17605 / OSF.IO / 7RB95).

### 2.2 Ethics

Since the scheme does not require patient recruitment and personal information collection, it does not require approval from an ETHICS committee.

### 2.3 Eligibility criteria

#### 2.3.1 Types of studies

We will collect all the randomized controlled trials of metformin in the treatment of gestational diabetes mellitus, regardless of blinding, publication status, region, but Language will be restricted to Chinese and English.

#### 2.3.2 Research object

The patients were clearly diagnosed as gestational diabetes,^[9]^ regardless of nationality, race, age, gender and course of disease.

#### 2.3.3 Intervention measures

The treatment group was treated by oral metformin alone, and studies combining other therapies were excluded. The control group was treated with insulin injection. The dosage, frequency and course of treatment were not restricted between the treatment group and the control group.

#### 2.3.4 Outcome index

(1) outcome indicators of pregnant women: fasting plasma glucose, 2 hour postprandial blood glucose, Hemoglobin a1c, Weight gain of pregnant women, incidence of cesarean section and preterm delivery during intervention;
(2) Outcome indicators of newborns: incidence of hypoglycemia, macrosomia, hyperbilirubinemia, and acute respiratory distress syndrome

### 2.4 Exclusion criteria

(1) Repeatedly published papers;
(2) articles in which the published literature is an abstract, the data is incomplete or the data is incorrect, and the complete data cannot be obtained after contacting the author;
(3) Literature that is assessed as a high risk of bias by randomizing or allocating concealment;^[14]^
(4) the mode of intervention is not consistent (including those treated with metformin in combination with other drugs and switching to insulin due to poor blood glucose control);
(5) Literature without relevant outcome indicators.

### 2.5 Retrieval strategy

“Gestational diabetes”(renshenqitangniaobing), “metformin” (erjiashuanggua) and “Gehua zhi” were used as Chinese search terms to search in Chinese databases, including China National Knowledge Network, Wanfang Data Knowledge Service Platform, Vipu Information Chinese Journal Service Platform, and China Biomedical Database. Gestational Diabetes Mellitus, “Gestational Diabetes Mellitus,” “GDM”“metformin,” “glucophage” are retrieved in English language databases including PubMed, EMBASE, Web of Science, and the Cochrane Library. The retrieval time was from the establishment of the database to November 2020, and all domestic and foreign literatures on metformin in the treatment of gestational diabetes were collected. Take PubMed as an example, and the retrieval strategy is shown in Table 1.

**Table 1:** Retrieval strategy of PubMed.

Number	Search terms
#1	Metformin [MeSH]
#2	Dimethylbiguanidine [Title/Abstract]
#3	Dimethylguanylguanidine [Title/Abstract]
#4	Glucophage [Title/Abstract]
#5	Metformin Hydrochloride [Title/Abstract]
#6	Metformin HCl [Title/Abstract]
#7	#1 OR #2 OR #4 OR #5 OR #6
#8	gestational diabetes [MeSH]
#9	Diabetes, Pregnancy-Induced [Title/Abstract]
#10	Pregnancy-Induced Diabetes [Title/Abstract]
#11	Diabetes Mellitus, Gestational [Title/Abstract]
#12	Gestational Diabetes Mellitus [Title/Abstract]
#13	#8 OR #9 #10 OR #11 OR #11 OR #12
#14	#7 AND #13

### 2.6 Data screening and extraction

**Figure 1:**
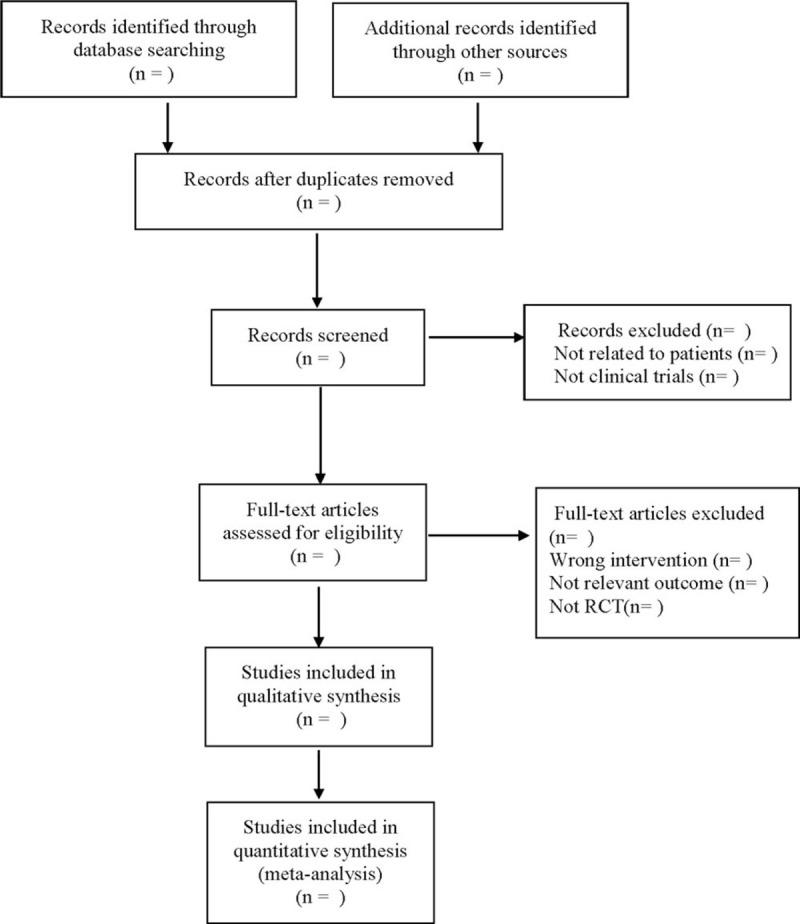
Flow diagram.

The data will be extracted independently by 2 researchers, the information will be recorded in the data extraction table, and if they have differences, it will be resolved with the help of a third reviewer. Detailed extraction information is as follows: ①Clinical study (title, first author, publication date, sample size, sex ratio, average age, average course of disease); ②Intervention measures (dosage form, frequency, course of treatment and follow-up time of the treatment group and control group); ③ Risk bias assessment factors in randomized controlled trials; ④Outcome index. The literature selection process is shown in Figure 1.

### 2.7 Literature quality assessment

The quality evaluation of the literature was completed by 2 researchers independently according to the Cochrane5.1.0 systematic evaluation manual, and the third researcher participated in the discussion when there was disagreement, and finally the overall quality of the literature was determined. The evaluation indicators include random sequence generation, allocation concealment, blind method, integrity of result data, selective reporting of research results and other sources of bias. According to these indicators, the included literature is evaluated as “high risk of bias,” “low risk of bias” and “unknown.”

### 2.8 Statistical analysis

#### 2.8.1 Data analysis and processing

The RevMan 5.3 software was used for Meta analysis. *X*^*2*^ and *I*^*2*^ were used to determine whether there was heterogeneity in each literature, and the heterogeneity of the effect value was analyzed. If *P* > .1, *I*^2^ < 50%, indicating low inter-study heterogeneity, fixed model was used for analysis. If *P* < .1 and *I*^2^≥50%, it indicates that there is significant heterogeneity among studies. The source of heterogeneity was analyzed and the random effect model was used for analysis. The measurement data were represented by the weighted mean difference or the Standard mean difference and 95%CI, and the counting data are represented by relative risk ratio and 95%CI.

#### 2.8.2 Dealing with missing data

If the data of the required study is incomplete or not reported in the study, the researcher will contact the first author or other author of the study by phone or email. If the required data are not available, we will use descriptive analysis instead of meta analysis and exclude these studies if necessary.

#### 2.8.3 Subgroup analysis

We will conduct a subgroup analysis based on the dosage, duration, and follow-up time of metformin after treatment.

#### 2.8.4 Sensitivity analysis

In order to test the stability of meta-analysis results of indicators, a 1-by-1 elimination method will be adopted for sensitivity analysis.

#### 2.8.5 Assessment of reporting biases

Funnel plots were used to assess publication bias if no fewer than 10 studies were included in an outcome measure. Moreover, Egger and Begg test were used for the evaluation of potential publication bias.

#### 2.8.6 Evidence quality evaluation

We will use the Grading of Recommendation Assessment, Development and Evaluation scoring method to grade the evidence of the outcome index.^[15]^ The evaluation content includes bias risk, indirectness, inconsistency, inaccuracy and publication bias, and the quality of evidence will be rated as high, medium, low or very low.

## 3 Discussion

Gestational diabetes mellitus (GDM) is a complication of abnormal glucose metabolism during pregnancy, which often occurs in the middle and third trimester of pregnancy, and the pathogenesis is unclear, which may be related to the increased secretion of progesterone, estrogen and other substances in the second and third trimester of pregnancy, resulting in increased insulin resistance, resulting in the decrease of maternal peripheral tissue sensitivity to insulin.^[16]^ GDM is not only associated with an increased risk of other complications during pregnancy, but also poses a long-term risk to mothers and their offspring ^[17]^

For GDM patients with ineffective diet and exercise control, insulin injection is the preferred treatment.^[9]^ Due to defects such as subcutaneous injection, inconvenient storage, high price and easy to lead to hypoglycemia, patients have poor compliance. While metformin, as an oral hypoglycemic drug, has a higher medical compliance. Metformin belongs to biguanide drugs, which can act on the liver, inhibit gluconeogenesis, reduce liver sugar output,^[18]^ and also act on peripheral tissues, reduce free fatty acids, promote muscle glycogen synthesis, increase glucagon-like peptide 1 in intestinal cells, and inhibit glucose absorption by intestinal parietal cells.^[19]^ Some studies have found that the content of metformin in the placenta is similar to that of the mother, so it is speculated that metformin can pass through the placental barrier.^[20]^ Therefore, there is still controversy on the feasibility of treating GDM, but there is no report on the adverse effect of metformin on neonatal outcomes.

A study of 3928 pregnant women with GDM (metformin 1996, insulin group 1932) and born children^[21]^ compared the long-term prognosis of school-age children treated with metformin and insulin for GDM. The results showed that there was no significant difference in growth and development assessment between metformin-treated mothers and insulin-treated mothers. A prospective study showed that^[22]^ average blood glucose levels, maternal weight gain during pregnancy and the incidence of neonatal hypoglycemia in GDM patients treated with metformin were significantly lower than those in GDM patients treated with insulin. Although a number of clinical studies have confirmed the efficacy and safety of metformin in patients with GDM, there are differences among studies. This study will evaluate the efficacy of metformin in patients with GDM based on current evidence, and evaluate its long-term effects on patients and newborns.

However, this systematic review has some limitations. There may be some clinical heterogeneity due to different drug doses, dosage forms and patients’ disease degrees among the included studies. Due to the different follow-up time of different studies, there are differences in the evaluation of long-term efficacy and safety. Due to the limitation of language retrieval, we will only include Chinese and English literature, and may ignore studies in other languages and regions.

### 3.1 Uncited reference

^[6]^.

## Author contributions

**Data collection**: Weirong Mao and Lanying Wang

**Funding support**: Shaohua Shen

**Literature retrieval**: Shengzhi Zhang and Lanying Wang

**Software operating**: Shengzhi Zhang

**Supervision**: Lanying Wang

**Writing – original draft**: Weirong Mao and Shengzhi Zhang

**Writing – review & editing**: Weirong Mao and Shaohua Shen
